# The First Complete Mitochondrial Genome of *Corydalis impatiens* (Papaveraceae) and Its Phylogenetic Implications

**DOI:** 10.3390/cimb48030291

**Published:** 2026-03-09

**Authors:** Qi’en Li, Digao Wan, Guixiang Wang, Xiuying Lin, Jiuli Wang, Huan Wang

**Affiliations:** 1 Tibetan Medical College, Qinghai University, Xining 810016, China; qienli2011@163.com (Q.L.); ag_gsgnzn_415@163.com (D.W.); 2 Qinghai Provincial Key Laboratory of High Value Utilization of Characteristic Economic Plants, Qinghai Minzu University, Xining 810007, China; wgx20021001@163.com (G.W.); lxy0105200607@outlook.com (X.L.); 3 Key Laboratory of Resource Chemistry and Eco-Environmental Protection in Qinghai-Tibet Plateau, Qinghai Minzu University, Xining 810007, China; 4 State Key Laboratory of Tibetan Medicine Research and Development, Qinghai University, Xining 810016, China; 5 College of Pharmacy, Qinghai Minzu University, Xining 810007, China

**Keywords:** mitochondrial genome, *Corydalis impatiens*, Papaveraceae, Ranunculales

## Abstract

*Corydalis impatiens* (Papaveraceae) is a traditional Tibetan medicinal plant (“Pa Xia Ga”) whose mitochondrial genome evolution remains unexplored, particularly in the context of high-altitude adaptation. This study presents the first complete mitochondrial genome sequence of an alpine *Corydalis* species to establish a comparative framework with the lowland congener *C. pauciovulata* for investigating environment-associated mitochondrial evolution. Using Illumina sequencing and reference-guided assembly, we characterized a 688,959 bp circular genome containing 74 genes, with GC content variations reflecting functional compartmentalization—elevated in structural RNA genes (tRNAs: 51.24%; rRNAs: 52.79%) versus protein-coding genes (44.19%). We identified 719 RNA editing sites concentrated in NADH dehydrogenase genes, suggesting post-transcriptional optimization of respiratory complex I under hypoxic conditions. The genome harbors 50 dispersed repeats (7.50%) and 67 SSRs with A-rich predominance, providing species-specific markers for authenticating “Pa Xia Ga” in Tibetan medicine quality control. Phylogenomic analysis confirms close affinity with *C. pauciovulata* while resolving intrageneral relationships within Ranunculales. These findings establish a dual-reference system for distinguishing conserved genus-level features from altitude-associated adaptations, enabling future comparative mitogenomics across the 465-species genus and supporting DNA-based medicinal plant identification.

## 1. Introduction

Mitochondria are semi-autonomous organelles that play a pivotal role in eukaryotic cells, including plants [1]. They are remnants of an ancient endosymbiotic event in which a proto-eukaryotic cell engulfed a bacterium, leading to the formation of an organelle with its own genetic material, distinct from the nuclear genome [2,3]. Plant mitochondria are central to various cellular processes such as respiration, energy metabolism, and programmed cell death, underscoring their importance in plant biology and agriculture [4,5]. Mitochondria in plants, as in all eukaryotes, retain their own genome (mitochondrial genome, mt genome), which is distinct from the nuclear genome and is a remnant of their endosymbiotic origin from ancient α-proteobacteria [6]. The mt genome in plants exhibits unique characteristics that set it apart from its animal counterparts, including a larger genome size, complex genomic arrangements, and a higher content of non-coding regions and introns [7,8]. These features render the study of plant mt genomes not only a fascinating exploration into the evolution of endosymbiotic organelles but also a gateway to understanding the genetic basis of important biological traits, while simultaneously supplying high-resolution markers for species delimitation and authenticated identification and offering genome-wide metrics to guide the conservation and management of endangered or economically valued plants.

The genus *Corydalis* (Papaveraceae), comprising over 465 species, represents the largest genus in the poppy family and includes numerous medicinal plants distributed across diverse habitats from sea level to high mountains [9]. Despite the availability of complete plastid genomes for multiple *Corydalis* species, which have revealed dynamic evolutionary changes including gene losses and inverted repeat boundary shifts [10,11,12], the mitochondrial genomes of this genus remain virtually unexplored. Recently, Park et al. (2024) [9] reported the first complete mitochondrial genome of *C. pauciovulata*, providing a critical baseline for understanding *Corydalis* mt genome architecture. However, this single lowland reference is insufficient to capture the evolutionary diversity of the genus, particularly for species inhabiting extreme environments. *Corydalis impatiens*, also known as “Pa Xia Ga”, is a traditional Tibetan medicinal plant that has been used for its analgesic, anti-inflammatory, and hepatoprotective properties [13]. It is a rich source of isoquinoline alkaloids, which exhibit a range of pharmacological activities, including antitumor effects [14]. Although the mitochondrial genome does not directly encode the enzymes for these valuable metabolites, it supplies the essential ATP and NAD(P)H required for their biosynthesis. As a congeneric species occupying a dramatically different ecological niche compared to *C. pauciovulata*, *C. impatiens* offers an opportunity to investigate how mitochondrial genomes evolve in response to high-altitude environmental pressures—specifically hypoxia, high UV radiation, and temperature extremes. The substantial geographic and ecological divergence between these two *Corydalis* species provides a natural experiment to identify environment-associated mitochondrial adaptations versus conserved genus-level features. Furthermore, the complete mt genome of *C. impatiens* will enable the development of DNA-based authentication markers to distinguish “Pa Xia Ga” from morphologically similar adulterants, addressing an urgent need in Tibetan medicine quality control [1].

In this study, we present the first complete mt genome sequence of *C. impatiens*, delving into its structure, coding content, and organizational diversity. This study establishes a new complete mitochondrial genome reference for the genus *Corydalis* and the first from an alpine environment. Specifically, the repeat markers identified here enable immediate DNA-based authentication of “Pa Xia Ga” to prevent adulteration in the Tibetan medicine trade, while the complete genome provides a necessary reference for comparative mitogenomic analyses across the 465-species genus. These resources address current gaps in medicinal plant quality control and phylogenetic studies of Papaveraceae.

## 2. Materials and Methods

### 2.1. Plant Sample Collection and DNA Extraction

Following the method used by Wan et al. (2022) [15], samples were collected and specimens were preserved. Fresh young leaves of Corydalis impatiens were gathered from Huangzhong County, Qinghai Province, China, at coordinates 36.27 N, 101.68 E. The voucher specimen (accession number LQE-2020-070) has been deposited in the Specimen Room of the Tibetan Medicine Research Center at Qinghai University (https://zyxy.qhu.edu.cn/jgsz/jxkysw/zyyyjzx/index.htm (accessed on 10 January 2026), contact: Qien Li, email: qienli@qhu.edu.cn). Total DNA was extracted from the silica gel-dried young leaves using a Plant Genomic DNA Kit (catalog number DP305, TIANGEN Biotech (Beijing) Co., Ltd., Beijing, China).

### 2.2. Sequencing and Quality Control

The second-generation sequencing experimental procedures were conducted in accordance with the standard protocol provided by Illumina [16], encompassing sample quality assessment, library construction, library quality assessment, and sequencing. Once the genomic DNA of the samples was verified to be of sufficient quality, ultrasonication was employed to fragment the DNA. Subsequent to fragmentation, the DNA was subjected to purification, end repair, addition of adenine to the 3′ ends, and ligation of sequencing adapters. Agarose gel electrophoresis was utilized to select fragments of appropriate size, and PCR amplification was carried out to form the sequencing library. The constructed libraries were initially assessed for quality, and those that passed the quality checks were sequenced using the Illumina Novaseq 6000 platform.

Sequencing generated a total of 17.2 Gb raw data, comprising 56.7 million raw reads (150 bp paired-end). Raw data were filtered using the fastp software (version 0.20.0, available at https://github.com/OpenGene/fastp (accessed on 10 January 2026)) [17] with the following criteria: (1) trimming of sequencing adapters and primer sequences from the reads; (2) filtering out reads with an average quality score below Q5; and (3) filtering out reads with more than five ambiguous nucleotides (Ns). After quality filtering, 52.5 million clean reads were retained, with Q30 = 91.8% and GC content of 38.8%.

### 2.3. Mt Genome Assembly

To mitigate the complexity of sequence assembly, we employed bowtie2 (version 2.2.4) [18] in the very-sensitive-local mode for alignment against reference sequences, utilizing the aligned sequences for subsequent assembly. The assembly process was conducted using the SPAdes software (version 3.10.1) [19] with default parameters. Following assembly, bowtie2 was again utilized to map the sequencing reads to the assembled contig sequences, identifying connectivity within the contigs. The sequences were then concatenated, and the resulting sequences were manually inspected and corrected to obtain a complete circular mt genome sequence. This approach, following the reference-guided strategy established for *Brassica* mitochondrial genomes [20], ensured the accuracy and integrity of the mt genome assembly.

### 2.4. Mt Genome Annotation

The annotation of the mt genome was conducted through a systematic approach as follows: (1) Protein-coding genes and rRNA genes were annotated by aligning against published plant mt sequences referenced in the database using BLAST v2.6 (https://blast.ncbi.nlm.nih.gov/Blast.cgi (accessed on 10 January 2026)) [21]. (2) tRNA genes were identified using the tRNAscan-SE tool (http://lowelab.ucsc.edu/tRNAscan-SE/ (accessed on 10 January 2026)) [22]. (3) RNA editing sites were predicted using the online resource PREP (https://bio.tools/prep_suite (accessed on 2 January 2026)) [23]. After rigorous examination and manual correction of the results, the final annotations were obtained.

### 2.5. Repeat Sequences Analysis

#### 2.5.1. Dispersed Repeat Sequences Analysis

Repetitive sequence identification was performed using vmatch (the only available release version) software (http://vmatch.de/ (accessed on 10 January 2026)). The parameters for this analysis were set as follows: a minimum length of 30 base pairs and a hamming distance of 3. The identification encompassed two types of repeats: direct (D) and palindromic (P).

#### 2.5.2. SSR Recognition and Analysis

The identification and analysis of simple sequence repeats (SSRs) within the mt genome of *C*. *impatiens* were performed utilizing MISA [24]. The parameters were configured as follows: the definition of unit size and minimum number of repeats were set to 1-10, 2-6, 3-5, 4-5, 5-5, and 6-5, respectively. Interruptions, defined as the maximum difference between two SSRs, were allowed up to 100 base pairs.

#### 2.5.3. Tandem Repeats Recognition

Analysis of tandem repeats in the mt genome of *C. impatiens* was conducted using the Tandem Repeats Finder program [25]. The parameters employed were as follows: match probability = 2, mismatch probability = 7, indel probability = 7, match score = 80, mismatch penalty = 10, indel penalty = 50, and maximum period size = 500.

### 2.6. Analysis of Codon Preference

The unique coding sequence (CDS) data of the mt genome were compiled and subsequently analyzed using CodonW version 1.4.2 to calculate the relative synonymous codon usage (RSCU) values [26]. The results were then graphically represented as a bar chart for visualization.

### 2.7. Nucleotide Diversity Analysis

Homologous protein coding gene sequences from the mt genomes of *C. impatiens* and other species within the order Ranunculales were subjected to global alignment using MAFFT (version 7) software in its automatic mode [27]. Subsequently, the dnasp6 program was employed to compute the pairwise nucleotide diversity (Pi) values for each gene [28]. The resulting π values were then plotted as a line graph for visualization.

### 2.8. Homology Analysis Between Mt and Cp Genomes

To assess the sequence homology between the mt and chloroplast genomes, we employed BLAST software to identify homologous sequences between the two organellar genomes, with a threshold set for sequence similarity at 70% and an E-value cutoff at 10^−5^ [21]. Following the identification of homologous sequences, Circos (version 0.69-10) was utilized to graphically represent the data [29].

### 2.9. Phylogenetic Analysis

Full mt genomes of *C. impatiens* and its closely related taxa within the order Ranunculales were downloaded from the NCBI GenBank database. Additionally, the complete mt genome of *Paeonia lactiflora*, belonging to the family Saxifragaceae, was obtained to serve as an outgroup.

A total of 21 conserved protein-coding genes shared across all analyzed species were selected for phylogenetic reconstruction: *mttB*, *ccmC*, *nad3*, nad6, *ccmFn*, *ccmFc*, *atp6*, *nad1*, *ccmB*, *atp9*, *nad7*, *nad4*, *rpl16*, *cox2*, *rps4*, *nad4L*, *atp4*, *cox1*, *matR*, *nad2*, *cox3*, and *atp8*. These genes were selected based on two criteria: (i) universal presence across all analyzed Ranunculales species and (ii) absence of RNA editing sites or minimal editing to avoid alignment artifacts [30]. MAFFT (version 7) was used to align the conserved coding sequences of these genomes [27], and MEGA (version 11) was employed for phylogenetic tree construction using the maximum-likelihood method with the Tamura–Nei model [31].

## 3. Results

### 3.1. Basic Characteristics of the C. impatiens Mt Genome

**Figure 1:**
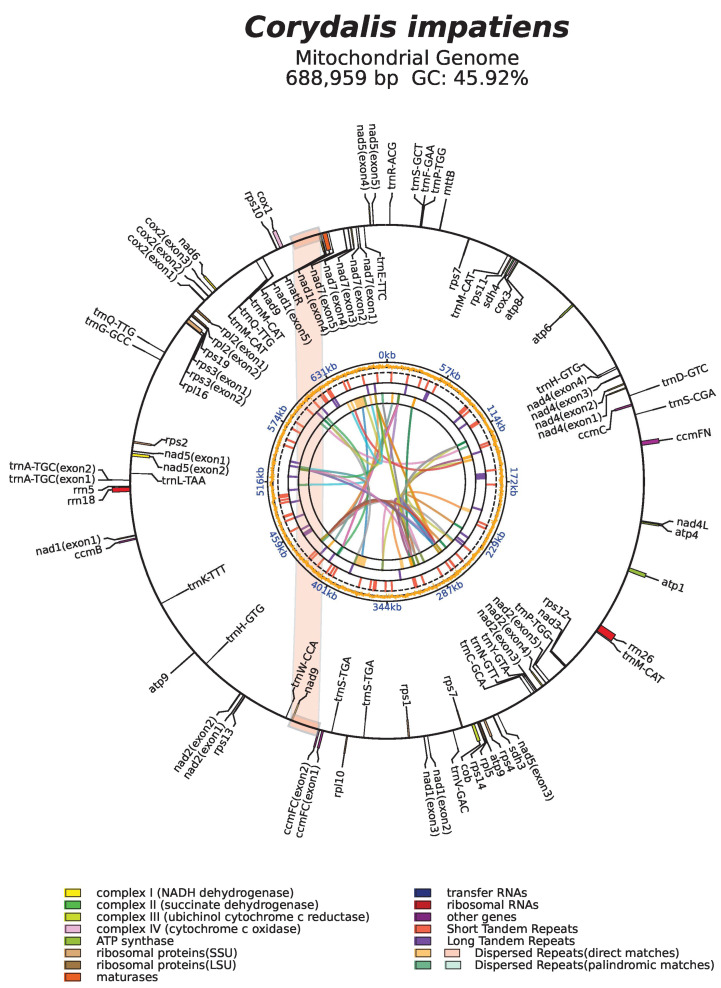
The mt genomic map. Genes belonging to different functional categories are depicted on the outer circle and color-coded for distinction: complex I (NADH dehydrogenase), complex II (succinate dehydrogenase), complex III (ubiquinol cytochrome c reductase), complex IV (cytochrome c oxidase), ATP synthase, small subunit (SSU) ribosomal proteins, large subunit (LSU) ribosomal proteins, maturases, transfer RNAs (tRNAs), ribosomal RNAs (rRNAs), and other functional genes. The color blocks on the outer circle represent long dispersed repeats. The inner circular layers display genomic structural features in turn, short tandem repeats, long tandem repeats, direct match dispersed repeats, and palindromic match dispersed repeats, with colored parabolas in the central circle highlighting the distribution and linkage of dispersed repeats across the genome.

The *C. impatiens* mt genome (NCBI accession number: PQ628113) is circular in structure with a total length of 688,959 bp and 74 genes (27 transfer RNAs (tRNAs), 3 ribosomal RNAs (rRNAs), and 44 protein-coding genes (PCGs); Figure 1, Table 1). Detailed characteristics of each gene can be obtained from Supplementary Table S1. The mt genome of *C. impatiens* exhibits GC content of 45.92%. In contrast to the mt genome, the PCGs display slightly lower GC content of 44.19%, indicating a modest decrease in the proportion of guanine and cytosine residues compared to the overall mt genome. The tRNAs have a higher GC content of 51.24%, suggesting a relative enrichment of guanine and cytosine in these genes responsible for protein synthesis. Lastly, the rRNAs show a GC content of 52.79%, which is the highest among the four categories, pointing to a significant compositional bias towards guanine and cytosine in the genes involved in ribosome structure and function (Table 2).

**Figure 2:**
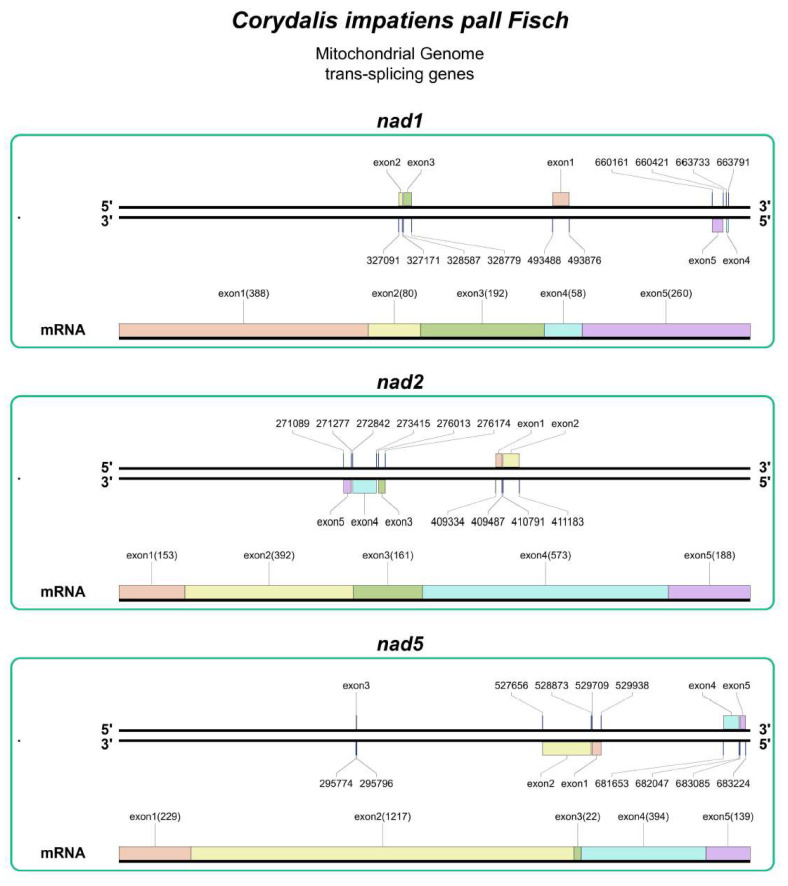
Trans-splicing gene map of the mt genome. The map displays three core trans-splicing genes (*nad1*, *nad2*, and *nad5*) of the plant mitochondrial genome, with each gene presented as discrete exons distributed on different genomic strands. For each exon, the corresponding genomic start and end positions are indicated following the exon label, and the length (bp) of each exon is shown in parentheses below the exon name. The mRNA line at the bottom of each gene represents the canonical 5′ → 3′ arrangement of exons after trans-splicing, reflecting the post-transcriptional assembly of discontinuous exons into functional messenger RNA. All trans-splicing genes are ordered by the genomic start position of their first exon.

**Figure 3:**
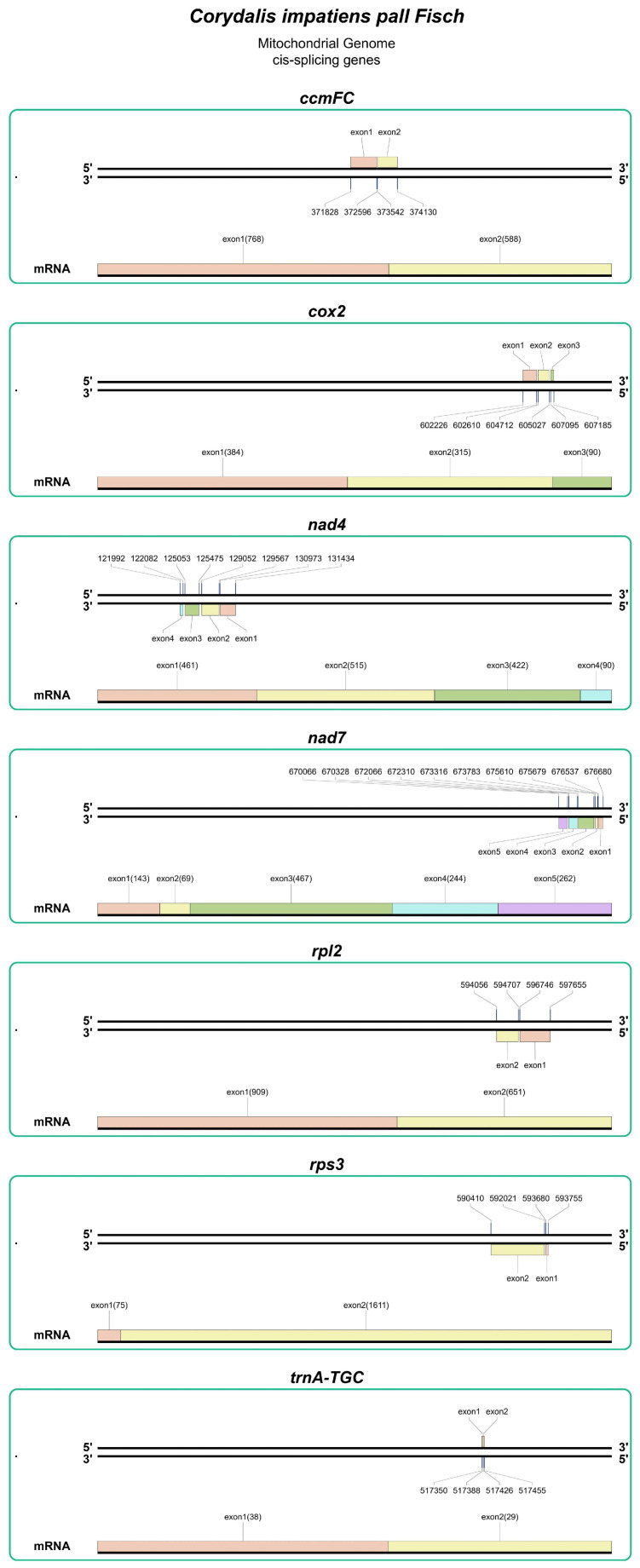
Cis-splicing gene map of the mt genome. The map presents seven core cis-splicing genes (*ccmFC*, *cox2*, *nad4*, *nad7*, *rpl2*, *rps3*, and *trnA*-*TGC*) of *C. impatiens* mitochondrial genome, with each gene displayed as discrete exons; the genomic start and end positions of each exon are indicated above the corresponding exon label, and the length (bp) of each exon is shown in parentheses following the exon name. The *mRNA* line at the bottom of each gene depicts the canonical 5′ → 3′ arrangement of exons after cis-splicing, reflecting the post-transcriptional assembly of exons from the same genomic strand into functional messenger RNA. All cis-splicing genes are organized with exons ordered according to their natural genomic distribution and splicing orientation.

Among the genes within the mt genome of *C. impatiens*, there are 10 genes that contain introns, specifically *ccmFC*, *nad1*, *nad2*, *nad4*, *nad5*, *nad7*, *rps3*, *rpl12*, *cox2*, and *trnA-TGC*. Notably, the subunits of the NADH dehydrogenase genes account for the highest number of introns, with a total of 19 introns present. Furthermore, within the mt genome, there are three trans-splicing genes (*nad1*, *nad2*, and *nad5*; Figure 2) and seven cis-splicing genes (*ccmFC*, *nad4*, *nad7*, *rps3*, *rpl12*, *cox2*, and *trnA-TGC*; Figure 3). The presence of the three trans-splicing genes indicates complex post-transcriptional processing in *C. impatiens*. This conserved feature across *Corydalis* species suggests essential regulatory mechanisms for respiratory complex I assembly, potentially enhancing metabolic flexibility in variable alpine environments. This intron distribution pattern, particularly the concentration of trans-splicing events in NADH dehydrogenase genes, reflects the complex post-transcriptional regulation required for respiratory complex I assembly in plant mitochondria.

### 3.2. RNA Editing Sites

RNA editing modulates the expression and stability of genes at the RNA level by altering nucleotide bases, as well as by introducing insertions or deletions [32]. This process is crucial for enhancing transcriptome diversity and expanding the proteomic repertoire within a cell [33,34]. Our analysis of the mt genome of *C. impatiens* has revealed a complex pattern of RNA editing events that are crucial for the post-transcriptional modification of gene products.

**Figure 4:**
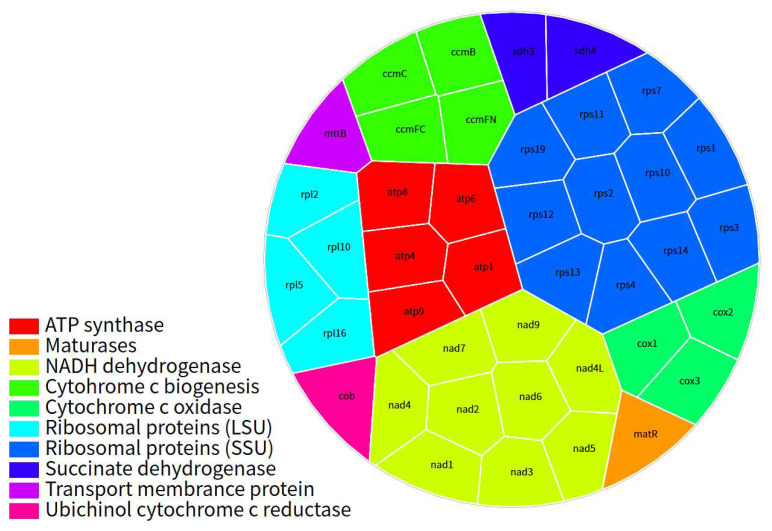
Voronoi TreeMap visualization of RNA editing site distribution across functional protein-coding genes in the mt genome. Each polygonal cell represents an individual protein-coding gene, with the cell area proportional to the number of RNA editing sites identified within that gene.

In the mt genome of *C. impatiens*, a total of 719 RNA editing sites have been predicted. Among these, the gene encoding NADH dehydrogenase (*nad4*) exhibits the highest number of editing sites, with 59 occurrences. In contrast, the genes encoding ribosomal protein S7 (*rps7*) and ribosomal protein S11 (*rps11*) have the fewest, each with only 2 editing sites. Collectively, the NADH dehydrogenase genes harbor the greatest number of RNA editing sites, a phenomenon attributed to the presence of as many as nine NADH dehydrogenase genes, with an average of 31 RNA editing sites per gene. The concentration of RNA editing in NADH dehydrogenase genes likely reflects selection for optimized electron transport chain function under hypoxic, high-UV conditions of the Qinghai–Tibet Plateau, consistent with adaptive metabolic reprogramming in alpine plants [35]. It is noteworthy that, despite comprising only four genes, the Cytochrome c biogenesis genes exhibit an exceptionally high average of 33.75 RNA editing sites per gene (Figure 4, Supplementary Table S2).

The confidence scores corresponding to these predictions vary between 0.23 and 1, with a significant proportion of edits possessing scores close to or equal to 1, indicating a high degree of reliability in our predictions.

### 3.3. Dispersed Repeats, Tandem Repeats, and Simple Sequence Repeats

**Figure 5:**
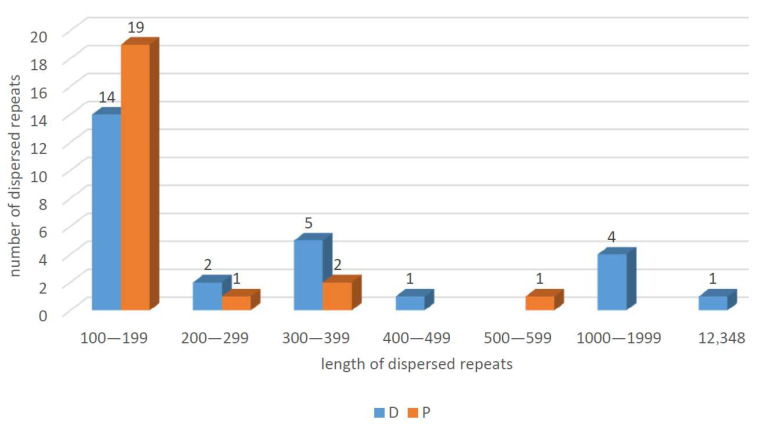
Dispersed repetitive sequences in the mt genome.

Dispersed repeat sequences are repetitive units that are present in a scattered form throughout the genome [36]. A total of 50 dispersed repeat sequences were detected in the *C. impatiens* mt genome, including 27 direct repeats (D) and 23 palindrome repeats (P), with repeat lengths mostly concentrated between 100 and 199 (33). The total length of the dispersed repetitive sequences is 51,640 bp, accounting for 7.50% of the total length of the mt genome. The distribution of dispersed repetitive sequences is shown in Figure 1. The length of each repeat sequence and the number of repeat types are shown in Figure 5 (detailed in Table S3).

Tandem repeats, which are sequences of 1 to 200 base pairs repeated consecutively, are a common feature in the genomes of eukaryotes and some prokaryotes A total of 47 tandem repeats were detected in the *C. impatiens* mt genome, with length distributions ranging from 9 to 150, and 24 tandem repeats had a match rate of >90%, as shown in Table 3. The distribution of repetitive sequences on the genome is also shown in Figure 1.

**Figure 6:**
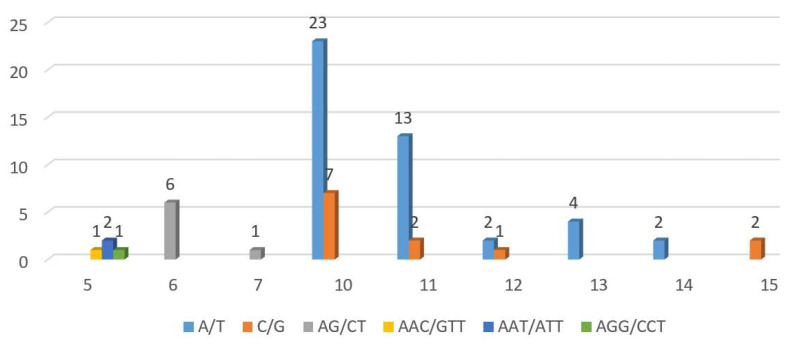
SSRs in the mt genome.

Simple sequence repeats (SSRs), comprising one to six nucleotide tandem repeats, are characterized by their high variability, reproducibility, multiallelic nature, abundance, and comprehensive genome coverage [37,38,39,40]. A total of 67 SSRs were detected in the *C. impatiens* mt genome, including 56 monomers, 7 dimers, and 4 trimers. The number of SSRs with A as a motif was the highest (44), accounting for 65.67% of the total number of SSRs (Figure 6).

These abundant repetitive elements, particularly the A-rich SSRs and dispersed repeats, provide species-specific molecular markers for authenticating “Pa Xia Ga” while potentially facilitating adaptive genome evolution through enhanced recombination capacity in the alpine environment.

### 3.4. RSCU of the Mt Genome

**Figure 7:**
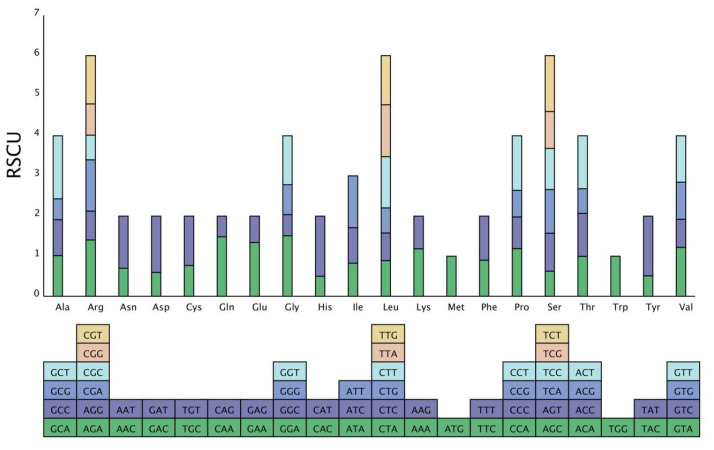
Relative synonymous codon usage (RSCU) histogram. The histogram displays the total RSCU value for each amino acid. The square below lists all synonymous codons for each amino acid, and each colored segment in the histogram bars corresponds to one of these codons, illustrating their individual contributions to the total RSCU.

The study of the mt genome codon preference of *C. impatiens* showed that 29 codons have relative synonymous codon usage (RSCU) > 1; of which 26 ended with A or T, accounting for 89.66%. In addition, the 87 bases that make up the 29 codons contain 24 A and 30 T bases, indicating that codons with this preference use more A/T bases in their composition. Thus; the *C. impatiens* mt genome has a significant AT preference. The schematic diagram of codon preference is shown in Figure 7. This AT bias aligns with the nucleotide composition of the *C. impatiens* mitochondrial genome and reflects the mutational signature prevalent in plant mitochondrial coding sequences.

### 3.5. Nucleotide Diversity

**Figure 8:**
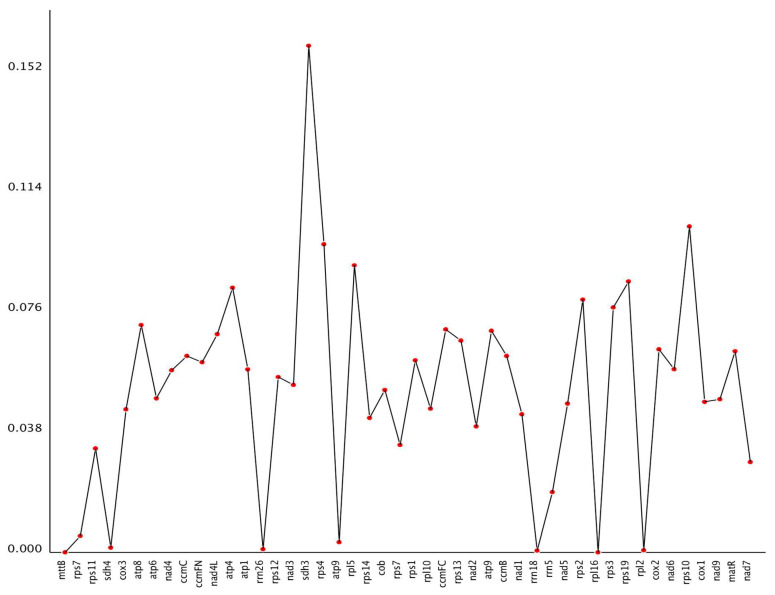
Nucleotide diversity (Pi) values for 46 mt genes.

Nucleotide diversity (Pi) across 46 protein-coding genes ranged from 0 to 0.15962 (Figure 8). The highest value was observed for *sdh3* (0.15962), 6.1-fold above the genome-wide mean (0.026), while *mttB* and *rpl16* showed complete conservation (Pi = 0). High diversity was also detected in ribosomal protein genes *rps4*, *rps10*, *rps19,* and *rpl5* (0.08333–0.10266), contrasting with their paralogues *rps7* (0.00513–0.03377) and conserved *rpl16*. Core respiratory genes exhibited intermediate values, with cytochrome c oxidase (0.04498–0.06389), NADH dehydrogenase (0.02836–0.06868) and ATP synthase (0.00317–0.08333) subunits spanning relatively narrow ranges. This variation in nucleotide diversity across genes may suggest differential selective constraints, with conserved genes maintaining essential respiratory functions and variable genes potentially accommodating adaptive changes.

### 3.6. Homology Analysis of Mt and Chloroplast Genomes

**Figure 9:**
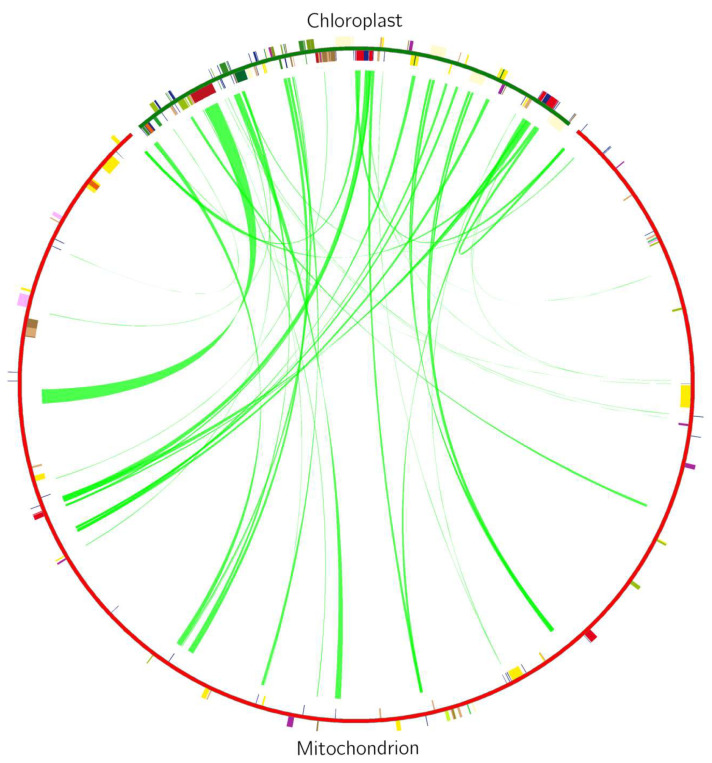
Distribution of homologous fragments in the cp and mt genomes of *C. impatiens*. The green segment of the circle represents the cp genome, and the red segment represents the mt genome. Genes from the same complex are labeled with the same color, and the middle line connection indicates homologous sequences.

Both the mt and cp genomes in *C. impatiens* were sequenced using the same tissue sample (leaf). Homology analysis revealed a transfer of DNA sequences from the cp genome to the mt genome. The mt and cp of *C. impatiens* contain a total of 47 shared fragments, ranging from 34 to 6493 bp in length, for a total length of 42,044 bp, or 6.10% of the total genome length (Figure 9, Table S4). This extent of sequence transfer indicates active intergenomic communication and contributes to the structural complexity observed in the *C. impatiens* mitochondrial genome.

### 3.7. Phylogenetic Relationships

**Figure 10:**
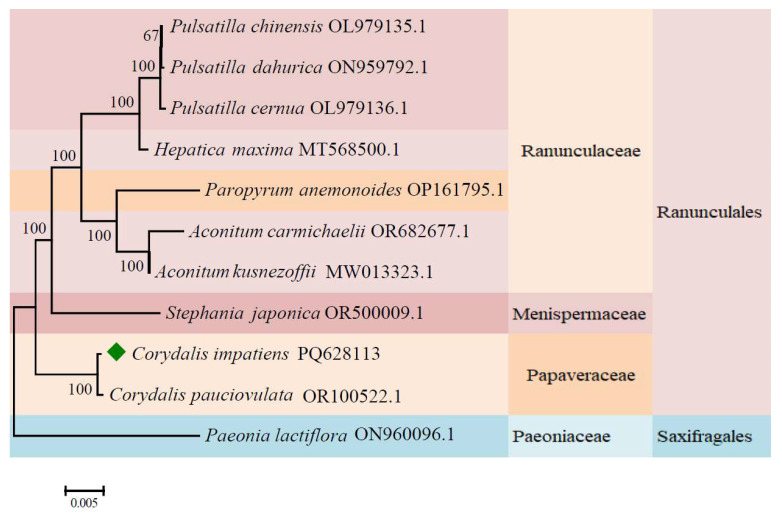
Phylogenetic relationships of *C. impatiens* with 10 other plant species. Maximum likelihood (ML) phylogenetic tree constructed using mitochondrial genome sequences of *Corydalis impatiens* Pall. and its closely related species from the Ranunculaceae and other associated families. The accession numbers of all mitochondrial genome sequences are indicated after the species names (e.g., *Corydalis impatiens* OR100522.1; *Corydalis pauciovulata* PQ628113). Bootstrap support values (100, 67, etc.) are labeled at the corresponding nodes, reflecting the statistical confidence of the phylogenetic clustering; a value of 100 indicates maximum support for the node. The scale bar at the bottom (0.005) represents the genetic distance (nucleotide substitution rate) among the species. The ◆ represents the genome sequences obtained in this study.

A phylogenetic tree was constructed based on the shared CDS sequences of the mt genomes of *C. impatiens* and nine other species from Ranunculales, with *Paeonia lactiflora* from Saxifragales serving as the outgroup. The tree showed that *C. impatiens* was the most genetically similar to *C. pauciovulata*. Except for the genus *Corydalis*, different species within the same genus on the phylogenetic tree each formed distinct clusters, and each family also grouped into relatively independent branches (Figure 10). This phylogenetic placement confirms the utility of mitochondrial genomes for resolving intrageneric relationships within *Corydalis*.

## 4. Discussion

Plant mitochondria are essential for energy metabolism, and their genomes are renowned for their dynamic nature, offering key insights into plant evolution and function [41]. The mt genome of plants is particularly valuable due to its variability, which includes a high content of repeated sequences and introns, and is subject to RNA editing [42]. This genetic plasticity is crucial for understanding adaptation and inheritance patterns in plants [41,42]. In this study, second-generation sequencing methods were used to study the mt genome of *C. impatiens*. The mt genome provides insights into the genetic architecture of this species.

### 4.1. Mt Genome Structure and Gene Composition

The mt genome of *C. impatiens* (688,959 bp, 74 genes) represents the second complete reference for the genus *Corydalis* and the first from an alpine environment. Comparison with *C. pauciovulata* (675,483 bp, 72 genes) [9] reveals both shared genus-level features and notable divergences. Both species retain identical core gene sets (44 PCGs, 3 rRNAs) and conserved trans-splicing patterns for *nad1/2/5*, indicating stable architectural constraints. However, *C. impatiens* exhibits a 13,476 bp size expansion driven by higher dispersed repeat content (7.50%). Despite this expansion, conserved gene order and intact coding sequences suggest robust genome stability mechanisms, possibly reflecting active DNA replication/repair processes as reported in *C. pauciovulata* [9] and functional constraints on essential respiratory genes.

The high GC content observed in tRNAs and rRNAs is intriguing, as it is a common feature in many mt genomes, potentially linked to the structural and functional requirements of these molecules. The lower GC content in protein-coding genes (PCGs) may reflect the diverse selective pressures acting on these genes, balancing the need for coding efficiency with the maintenance of protein function [43].

Practically, the species-specific repeat profiles and SSRs enable DNA-based authentication of “Pa Xia Ga,” addressing quality control needs in Tibetan medicine [44,45]. This dual reference system establishes a foundation for comparative mitogenomics in this pharmacologically important genus [9,11,12].

### 4.2. Intron Presence and Splicing Patterns

A total of 10 genes in the mt genome of *C. impatiens* contain introns, with a notable observation that half of these are NADH dehydrogenase (*nad*) genes. Moreover, the number of introns within the *nad* genes is also relatively high. The high number of introns in these genes may be associated with the complexity of their protein products and the need for post-transcriptional regulation [46]. The identification of cis- and trans-splicing genes in *C. impatiens* provides insight into the complexity of gene expression within plant mitochondria. This discovery also underscores the potential for alternative splicing events, which could contribute significantly to proteomic diversity [47,48]. The identical trans-splicing patterns between *C. impatiens* and *C. pauciovulata* [9], conserved across eudicots and basal angiosperms [48,49], indicate that this complex processing mechanism is under strong purifying selection rather than representing a recent adaptation. The maintenance of discontinuous gene structures for respiratory complex I subunits suggests ancient functional constraints on assembly efficiency, with limited scope for environmental modulation.

### 4.3. RNA Editing and Its Implications

The high number of RNA editing sites, totaling 719, in the mt genome of *C. impatiens* shows the significant role of RNA editing in the functioning of the *C. impatiens* mt genome. The concentration of editing events in *nad* genes suggests a critical role for RNA editing in fine-tuning the function of these proteins, possibly to adapt to specific metabolic demands or environmental stresses [50,51,52]. The concentration of editing sites in NADH dehydrogenase genes reflects the structural complexity of respiratory complex I, where C-to-U editing restores conserved hydrophobic residues essential for membrane insertion [32,51]. Comparative analysis with *C. pauciovulata* reveals comparable editing densities [9], suggesting that RNA editing in *Corydalis* is primarily constrained by protein functional requirements rather than representing a plastic response to hypoxia. Experimental validation of editing efficiency under stress would be required to test environmental modulation hypotheses.

### 4.4. Repeated Sequences and Their Genomic Impact

The presence of these repeats may contribute to genome plasticity and the potential for novel gene creation through mechanisms such as slipped-strand mispairing and unequal crossing over. The significant proportion of the genome composed of dispersed repeats (7.50%) also suggests a role in genome size variation and evolution.

The abundance of SSRs, particularly those with A as the motif, in the *C. impatiens* mt genome highlights the potential use of these regions as genetic markers [53]. The high number of A motifs in SSRs may reflect the overall AT-richness of the genome and could be indicative of the mutational bias towards A/T nucleotides [54].

The elevated repeat content in *C. impatiens* (7.50%) relative to the lowland congener *C. pauciovulata* (6.82%) [9] may reflect stochastic accumulation of non-coding DNA rather than adaptive selection for recombination capacity. Plant mitochondrial genomes exhibit extensive size variation driven by repeat proliferation without consistent correlation with environmental stress [7,8]. The absence of rearrangements in core protein-coding regions suggests that recombination is constrained by purifying selection on essential respiratory genes [7], consistent with the “structured variation” pattern observed across autotrophic angiosperms.

### 4.5. Codon Usage Bias

The mt genome of *C. impatiens* exhibits a significant bias towards codons ending in A or T, with 89.66% of the 29 most frequently used codons featuring these nucleotides. This preference suggests an adaptation that may optimize translation efficiency within the mitochondria. The high usage of A/T-rich codons could be due to the degeneracy of the genetic code and the potential for reduced reliance on G and C, which could be influenced by replication and transcription constraints in the organelle [55].

The AT-bias might also reflect the action of mutational pressures specific to the mitochondrial DNA polymerase, leading to a higher rate of A/T substitutions. This bias could impact translational accuracy and efficiency, potentially affecting protein expression levels within the mitochondria [56].

Understanding the codon usage bias in *C. impatiens* provides insights into the evolutionary pressures on plant mitochondrial gene expression and may reveal common selective forces across different plant species [57]. Further research is necessary to explore the functional implications of this bias on mitochondrial function and its role in plant evolution.

The AT-bias in *C. impatiens* (54.08% genome-wide) aligns with the documented mutational bias of plant mitochondrial DNA polymerases towards A/T substitutions [55,56]. This pattern is attributed to spontaneous cytosine deamination and replication errors rather than selection for metabolic efficiency. The comparable AT-content between *C. impatiens* and *C. pauciovulata* (reanalysis: 53.7%) [9] supports genus-level mutational pressures over altitude-specific adaptation.

### 4.6. Selective Constraints on Nucleotide Diversity

The nucleotide diversity (Pi) analysis within the mitochondrial genome of *C. impatiens* highlights significant variation among genes. The gene *sdh3*, involved in the electron transport chain, exhibits the highest Pi value (0.15962), suggesting it may be under unique selective pressures or experiencing higher mutation rates. Other genes such as *atp4*, *rps19*, *rpl5*, *rps4*, and *rps10* also show considerable diversity, indicating potential roles in adaptive evolution [58]. In contrast, *mttB* and *rpl16* display no diversity (Pi = 0), indicating that their conservation is likely due to functional constraints, as mutations in these essential genes could be detrimental. Their conservation indicates the importance of these genes in maintaining mitochondrial function [59,60].

The variable nucleotide diversity across genes (Pi = 0 to 0.15962) reflects differential selective constraints rather than uniform environmental adaptation. The conservation of *mttB* and *rpl16* (Pi = 0) indicates strong purifying selection on essential functions [61,62], while elevated diversity in *sdh3* (Pi = 0.15962) and ribosomal protein genes may reflect relaxed constraint or lineage-specific divergence. This pattern aligns with the “conserved core, variable periphery” model of plant mitochondrial evolution [8].

The Pi values reflect a balance between genetic variation and functional necessity within the *C. impatiens* mitochondrial genome, offering insights into the evolutionary dynamics of plant mitochondrial genes.

### 4.7. Homology Analysis and Gene Transfer

The comparison between the mt and cp genomes of *C. impatiens* reveals a complex interplay between these two organellar genomes. The presence of shared fragments between the two genomes, totaling 42,044 bp or 6.10% of the total genome length, suggests a degree of genetic exchange that could have implications for the understanding of genome evolution in plants [63,64]. Such intergenomic transfers are common in angiosperms and can lead to the transfer of genetic material between the organelles, potentially influencing their structure, function, and evolutionary trajectories [26,64].

This intergenomic transfer could have significant implications for our understanding of genome evolution and the potential for functional gene transfer between organelles.

### 4.8. Phylogenetic Insights

The phylogenetic tree based on the shared coding sequences (CDSs) of the mitochondrial genomes of *C. impatiens* and 10 other species provides valuable insights into the evolutionary relationships within Ranunculales. The tree constructed reveals that *C. impatiens* is most genetically similar to *C. pauciovulata,* indicating a close phylogenetic affinity between these two species.

The distinct clustering of different species within the same genus, along with the formation of relatively independent branches for each family, is consistent with the current Angiosperm Phylogeny Group IV (APG IV) system [65]. This indicates the usefulness of mitochondrial genomes in resolving phylogenetic relationships at various taxonomic levels. This pattern of genetic divergence suggests that the mitochondrial genome is a reliable molecular marker, reflecting the evolutionary history and divergence of these species.

The mt genome of *C. impatiens* offers a rich dataset for exploring genetic diversity, genome evolution, and the functional implications of mt gene expression. Our findings contribute to the broader understanding of plant mt biology and provide a foundation for further research into the adaptive significance of the observed genetic features.

### 4.9. Limitations and Perspectives

Despite establishing the first complete mitochondrial genome reference for an alpine *Corydalis* species and providing a comparative framework with the lowland congener *C. pauciovulata*, this study represents a single genome for a genus comprising over 465 species. This limitation constrains generalizability for testing altitude-associated evolutionary hypotheses across the genus. Future population-level resequencing of *C. impatiens* across its altitudinal range, together with additional mitogenomes from diverse *Corydalis* species, will be essential to validate the evolutionary patterns proposed herein.

## 5. Conclusions

The present study presents the first complete mitochondrial genome of an alpine *Corydalis* species, *C. impatiens*, establishing a critical reference for comparative mitogenomics in this pharmacologically important genus. Our analysis reveals that despite substantial ecological divergence between alpine *C. impatiens* and lowland *C. pauciovulata*, mitochondrial genome architecture remains largely conserved, with size variation driven primarily by stochastic accumulation of repetitive elements rather than altitude-specific adaptive processes. This finding underscores the importance of dual reference systems for distinguishing conserved genus-level features from environment-associated evolution. The identified species-specific SSR and repeat markers provide immediately applicable genetic tools for authenticating “Pa Xia Ga” in Tibetan medicine quality control, directly addressing the urgent need to combat adulteration in herbal markets. Furthermore, this genome resource enables future population-level studies of mitochondrial adaptation across the 465-species genus and supports phylogenetic resolution within Ranunculales. By integrating structural, functional, and comparative genomic analyses, this work advances our understanding of plant mitochondrial evolution while delivering practical resources for medicinal plant identification and conservation.

## Figures and Tables

**Table 1:** Genes present and functional gene category in the mt genome.

Group of Genes	Gene Name
ATP synthase	*atp1 atp4 atp6 atp8 atp9* (2)
Cytochrome c biogenesis	*ccmB ccmC ccmFC* * *ccmFN*
Ubichinol cytochrome c reductase	*Cob*
Cytochrome c oxidase	*cox1 cox2* ** *cox3*
Maturases	*matR*
Transport membrane protein	*mttB*
NADH dehydrogenase	*nad1* **** *nad2* **** *nad3 nad4* *** *nad4L nad5* **** *nad6 nad7* **** *nad9* (2)
Ribosomal large subunit (LSU)	*rpl10 rpl16 rpl2* * *rpl5*
Ribosomal small subunit (SSU)	*rps1 rps10 rps11 rps12 rps13 rps14 rps19 rps2 rps3* * *rps4 rps7* (2)
Succinate dehydrogenase	*sdh3 sdh4*
Ribosomal RNAs	*rrn18 rrn26 rrn5*
Transfer RNAs	*trnA-TGC* * *trnC-GCA trnD-GTC trnE-TTC trnF-GAA trnG-GCC trnH-GTG* (2) *trnK-TTT trnL-TAA trnM-CAT* (4) *trnN-GTT trnP-TGG* (2) *trnQ-TTG* (2) *trnR-ACG trnS-CGA trnS-GCT trnS-TGA* (2) *trnV-GAC trnW-CCA trnY-GTA*

Notes: The digit in brackets after the gene names indicates the number of gene copies. The number of asterisks (*) after the gene name represents the number of introns identified within a particular gene.

**Table 2:** The base composition of the mt genome.

Corydalis impatiens	Size (bp)	A%	T%	G%	C%	A + T%	G + C%	AT-Skew	GC-Skew
mt genome	688,959	27.03	27.05	22.97	22.95	54.08	45.92	0	0
PCGs	35,529	26.6	29.21	22.2	21.99	55.81	44.19	−0.047	0.005
tRNAs	2012	22.81	25.94	28.43	22.81	48.76	51.24	−0.064	0.11
rRNAs	4969	25.34	21.88	29.14	23.65	47.21	52.79	0.073	0.104

**Table 3:** Tandem repeat characteristics.

Indices	Period Size	Copy Number	Consensus Size	Percent Matches	Percent Indels	Score	Entropy (0–2)
21,949–21,997	24	2.2	20	77	19	53	1.64
46,113–46,152	21	1.9	21	84	0	53	1.64
48,089–48,129	18	2.3	18	95	0	73	1.8
92,823–92,891	15	4.3	15	75	17	59	1.96
92,807–92,870	18	3.6	18	80	4	67	1.97
93,041–93,136	21	4.6	21	73	10	79	1.93
103,053–103,096	21	2.1	21	82	0	52	1.94
138,073–138,122	24	2.1	24	100	0	100	1.94
163,528–163,576	24	2.1	24	92	3	82	1.88
164,362–164,437	15	4.8	16	75	24	56	1.91
164,362–164,445	9	10.5	8	72	27	72	1.91
164,362–164,446	24	3.5	24	100	0	170	1.91
164,462–164,782	24	13.1	24	82	5	329	1.98
164,446–164,761	150	2.1	150	85	2	420	1.98
165,071–165,215	48	3	48	96	0	263	1.97
165,492–165,544	18	2.9	18	86	5	72	1.92
165,586–165,684	30	3.3	30	82	0	126	1.99
165,680–165,796	36	3.6	36	68	25	123	1.98
165,711–165,820	12	9.2	12	81	8	109	1.97
165,684–165,836	60	2.5	60	100	0	306	1.97
166,904–166,950	20	2.3	20	96	0	85	1.96
168,348–168,433	22	3.9	22	95	0	154	1.88
204,152–204,191	21	1.9	21	84	0	53	1.84
238,566–238,608	18	2.4	18	81	18	54	1.96
245,628–245,681	21	2.6	21	96	0	99	1.97
248,860–248,922	33	1.9	33	83	0	81	1.45
329,306–329,351	22	2.1	22	95	0	83	1.91
387,089–387,130	19	2.2	19	95	0	75	1.58
413,516–413,555	18	2.2	19	86	13	57	1.6
426,136–426,165	15	2	15	93	0	51	1.8
453,449–453,477	14	2	15	93	6	51	1.98
455,776–455,813	20	2	19	85	10	51	1.97
460,922–460,971	25	2	25	100	0	100	1.87
475,712–475,744	15	2.2	15	100	0	66	1.85
493,396–493,474	39	2	39	95	0	140	1.98
507,258–507,291	18	1.9	18	94	5	61	1.97
522,522–522,575	26	2.1	26	89	0	81	1.97
534,425–534,511	18	4.8	18	98	0	156	1.91
539,316–539,378	10	6.2	10	74	3	54	1.98
539,430–539,524	50	1.9	50	97	0	181	1.94
602,110–602,175	29	2.3	29	100	0	132	1.9
609,727–609,769	22	1.9	23	90	4	70	1.66
624,428–624,459	15	2.1	16	94	5	57	1.88
627,427–627,464	19	2	19	100	0	76	1.93
666,325–666,361	12	2.7	15	76	24	53	1.96
674,947–674,975	14	2	15	93	6	51	1.13

## Data Availability

The genome sequence data obtained in this study are openly available in GenBank of the NCBI at https://www.ncbi.nlm.nih.gov/ (accessed on 14 January 2026) under the accession number PQ628113. The associated BioProject, SRA, and Bio-Sample numbers are PRJNA776704, SRX12869145, and SAMN22814974, respectively.

## Supplementary Materials

The following supporting information can be downloaded at: https://www.mdpi.com/article/10.3390/cimb48030291/s1.
